# Magnesium Application Promotes Rubisco Activation and Contributes to High-Temperature Stress Alleviation in Wheat During the Grain Filling

**DOI:** 10.3389/fpls.2021.675582

**Published:** 2021-06-11

**Authors:** Yuhang Shao, Shiyu Li, Lijun Gao, Chuanjiao Sun, Jinling Hu, Attiq Ullah, Jingwen Gao, Xinxin Li, Sixi Liu, Dong Jiang, Weixing Cao, Zhongwei Tian, Tingbo Dai

**Affiliations:** ^1^Key Laboratory of Crop Physiology Ecology and Production Management, Ministry of Agriculture and Rural Affairs, Nanjing Agricultural University, Nanjing, China; ^2^Rural Energy and Environment Agency, Ministry of Agriculture and Rural Affairs, Beijing, China; ^3^Chengdu Agricultural Technology Extension Station, Chengdu, China

**Keywords:** wheat, high-temperature stress, magnesium, Rubisco activity, Rubisco activation, photosynthetic rate, high-temperature stress, photosynthetic rate

## Abstract

Inhibited photosynthesis caused by post-anthesis high-temperature stress (HTS) leads to decreased wheat grain yield. Magnesium (Mg) plays critical roles in photosynthesis; however, its function under HTS during wheat grain filling remains poorly understood. Therefore, in this study, we investigated the effects of Mg on the impact of HTS on photosynthesis during wheat grain filling by conducting pot experiments in controlled-climate chambers. Plants were subjected to a day/night temperature cycle of 32°C/22°C for 5 days during post-anthesis; the control temperature was set at 26°C/16°C. Mg was applied at the booting stage, with untreated plants used as a control. HTS reduced the yield and net photosynthetic rate (P_*n*_) of wheat plants. The maximum carboxylation rate (V_*Cmax*_), which is limited by Rubisco activity, decreased earlier than the light-saturated potential electron transport rate. This decrease in V_*Cmax*_ was caused by decreased Rubisco activation state under HTS. Mg application reduced yield loss by stabilizing P_*n*_. Rubisco activation was enhanced by increasing Rubisco activase activity following Mg application, thereby stabilizing P_*n*_. We conclude that Mg maintains Rubisco activation, thereby helping to stabilize P_*n*_ under HTS.

## Introduction

Wheat is a C3 crop that grows during the winter and spring seasons, with an optimum grain filling temperature of 20–24°C (Paulsen, 1994; Shah and Paulsen, 2003; Li et al., 2012). The temperature threshold for grain filling in winter wheat is 25°C (Porter and Gawith, 1999; Wahid et al., 2007). When day/night temperatures increase to 30°C/25°C, the filling duration is shortened and dry matter accumulation decreases (Huebner and Bietz, 1988; Khan et al., 2020). However, the temperature often rises above 30°C in late spring, during the middle or late grain filling late stage, around the middle to lower reaches of the Yangtze River (World Weather Information Service). Grain yield can decrease by up to 30% at temperatures exceeding 32°C (Al-Khatib and Paulsen, 1990; Djanaguiraman et al., 2020). Global warming has increased the occurrence of intense high-temperature events (IPCC, 2020); as a result, high-temperature stress (HTS) on winter wheat during the grain filling stages restricts wheat production (Lesk et al., 2016).

Under favorable conditions, approximately 70–90% of the final grain yield is obtained from photosynthates produced during grain filling (Austin et al., 1977; Makunga et al., 1978; Chaudhuri et al., 2017). Longer leafing duration and higher photosynthetic activity contribute to yield increases in most major crops (Richards, 2000). However, high temperatures during post-anthesis restrict grain yield by reducing grain filling time and photosynthesis efficiency (Farooq et al., 2011). Photosynthesis is the basis of biomass production and is the physiological process that is most sensitive to elevated temperature, often showing inhibition before all other cellular functions at high temperatures (Berry and Bjorkman, 1980). Several photosynthetic pigment components of photosystem II (PSII), Rubisco carboxylation activity, stomatal opening, and other associated processes, are highly susceptible to HTS (Mathur et al., 2014). Damage to any of these components and processes is sufficient to interrupt the general photosynthetic mechanism (Ashraf and Harris, 2013; Mathur et al., 2014). However, the effects of HTS on the photosynthesis process during the wheat post-anthesis stage remain further understood.

Many studies have suggested that the loss of Rubisco activation is the main factor limiting the net photosynthesis rate under moderate HTS (Demirevska-Kepova and Feller, 2004; Salvucci and Crafts-Brandner, 2004b). Rubisco catalyzes the assimilation of CO_2_ during photosynthesis, and its catalytic limitations compromise photosynthesis efficiency (Parry et al., 2008; Lobo et al., 2019). Rubisco binds easily with inhibitors that block its active sites (Parry et al., 2008; Lobo et al., 2019). Rubisco activation state is defined as the fraction of active sites with catalytic activity and is regulated by Rubisco activase (RCA) (Boexfontvieille et al., 2014; Scafaro et al., 2016; Perdomo et al., 2017). Phosphate inhibitors can be removed from Rubisco active sites (carbamylated or not) via RCA activity in an ATP-dependent reaction (Streusand and Portis, 1987; Bracher et al., 2017). Under HTS, when Rubisco deactivation speeds up and photosynthesis is inhibited, the role of RCA becomes increasingly evident (Portis, 2003; Ristic et al., 2009; Scafaro et al., 2016; Perdomo et al., 2017). RCA is extremely sensitive to temperature (Kurek et al., 2007), but thermally stable RCA maintains high Rubisco activation levels and increases CO_2_ fixation efficiency (Kurek et al., 2007; Scafaro et al., 2016, 2019; Shivhare and Mueller-Cajar, 2017; Degen et al., 2020, 2021). Thus, improving RCA activity may be an effective method for maintaining a higher photosynthesis rate under HTS.

Magnesium (Mg) is involved in several physiological and biochemical processes of plant growth and development (Waraich et al., 2011). Up to 15–35% of total Mg in plants is located in chloroplasts (Karley and White, 2009; Chen et al., 2018), where it is involved in photophosphorylation and CO_2_ fixation (Cakmak and YaziCi, 2010). Mg mainly plays roles in Rubisco activation and RCA catalysis (Parry et al., 2008). Mg deficiency adversely affects CO_2_ fixation, leading to reduced photosynthesis rates (Andersson, 2008), whereas adequate Mg has been shown to alleviate adverse effects of HTS in wheat and maize seedlings (Mengutay et al., 2013). Thus, Mg may play a significant part in carbon reactions, representing a possible mechanism for stabilizing the net photosynthesis rate under HTS.

High-temperature stress frequently occurs during the grain filling stage; however, few studies have examined Mg functions involved in photosynthesis under HTS during the grain filling stage. We hypothesized that under HTS, Mg application would enhance Rubisco activation, contributing to HTS alleviation. Therefore, we examined variation in Rubisco content and Rubisco activation state, light energy utilization efficiency under HTS to evaluate the effects of Mg application on Rubisco carboxylation activity, and electron transport capacity under HTS.

## Materials and Methods

### Plant Culture and Growth Conditions

We conducted pot experiments at the Pailou Experimental Station of NAU, China (32°04′N, 118°76′E), during the 2016–2018 growing season. The local widely grown cultivar of Yangmai-16 (*Triticum aestivum* L.) was grown in plastic pots with a volume of 0.015 m^3^ (height, 30 cm; diameter, 25 cm), each with three holes at the bottom. Every pot was filled with 9 kg of air-dried and uniformly mixed clayey loam soil sieved through a 0.5-mm mesh. The soil contained 11.78 g kg^–1^ organic matter, 0.87 g kg^–1^ total nitrogen (N), 81.37 mg kg^–1^ available potassium (K), 19.25 mg kg^–1^ available phosphate (P), and 110.9 mg kg^–1^ available Mg. After soil filling, 5 g of compound fertilizer (15% N, 15% P, and 15% K) was applied to each treatment. At the jointing (Feekes 6.0) and booting stages (Feekes 10.0) (Miller, 1992), 0.45 g of N was applied to each pot. Each pot was sowed with 18 seeds, and then seedlings were scattered to eight in each pot at the three-leaf stage.

### Treatment Application and Management

Magnesium fertilizer in the form of MgSO_4_⋅7H_2_O was applied at 0.85 g per pot (0.11 g⋅kg^–1^) at the booting stage (Feekes 10.0), and pots without additional Mg application were used as controls (CK). The pots were moved to climate-controlled chambers set at a different temperature for 16–20 days after anthesis (DAA). HTS of the air temperature was simulated at 32°C/22°C, and 26°C/16°C was applied as the optimal temperature (OT). The temperature setting during treatment days and the leaf temperature are shown in Supplementary Figure 1. Relative humidity in the chambers was at 65%, under a light intensity of 1500 μmol photons m^–2^ s^–1^ with a photoperiod of 16 h. Each pot was watered about 2–4 l each morning and evening during post-anthesis. Following treatment, pots were relocated in the OT growth chamber until maturity. Thus, the four treatments were CK-OT (control fertilizer with optimal temperature), CK-HT (control fertilizer with HTS), Mg-OT (additional Mg fertilizer with optimal temperature), and Mg-HT (additional Mg fertilizer with HTS).

### Plant Sampling and Measurements

Plant sampling were conducted at 15DAA (prior to HTS treatment), 17DAA (2 days after treatment), 20DAA (5 days after treatment), and 25DAA. The flag leaves were detached from five randomly selected pots for each treatment and immediately submerged in liquid nitrogen for fresh sample measurements. Dry samples were collected from whole plants in five randomly selected pots of each treatment. We manually cut the plants at ground level using pruning scissors and then dried the samples at 105°C for 15 min in the oven, followed by 70°C until constant weight. The dried samples were ground for N and Mg content determination. Yield measurements at the maturity stage were tracked using the same five pots in each treatment.

### Gas Exchange and Fluorescence Measurements

#### Gas Exchange Measurements

The gas exchange parameters were measured in flag leaves using the red and blue light source chamber (LI6400-02B) of a gas exchange machine (Li-Cor 6400, Li-Cor Inc., United States) in this study. The gas exchange parameter measurements were according to the methods by Gao et al. (2018) with modifications. Measurements were performed from 9:00 to 11:00 am under a light level of 1500 μmol photons m^–2^ s^–1^. The vapor pressure deficit was between 0.5 and 1.0 kPa. The reference CO_2_ conference level was set at 400 μmol mol^–1^, and the relative humidity of the leaf chamber was set to 55–65%. The temperature of the leaf chamber was set according to real-time temperature in the growth chamber. The equipment was preheated for 0.5 h prior to measurement, and data were recorded three times after cuvette acclimatization for at least 5 min.

The net photosynthesis rate/intercellular CO_2_ concentration (A–C_*i*_) curve was determined according to Gao et al. (2018). Measurements were conducted using the red and blue light source chamber of the Li-Cor 6400 photosynthesis instrument. The setting of light level and relative humidity was the same with the previous setting. A CO_2_ injection system was used to control the CO_2_ concentration. Leaves were placed in the chamber for 10 min for adaptation. The CO_2_ concentration in the leaf chamber was then adjusted to values of 400, 200, 100, 50, 100, 150, 200, 400, 600, 800, 1000, 1200, and 1600 μmol mol^–1^, and data were recorded for about 3 min per setting. Plotting P_*n*_ as the vertical coordinate and C_*i*_ as horizontal coordinate, the initial slope of the curve (C_*i*_ < 200 μmol mol^–1^) represented carboxylation efficiency (CE). The Rubisco maximum carboxylation rate (V_*Cmax*_) and maximum electron transport rate (J_*max*_) were calculated according to modified equations (Long and Bernacchi, 2003; Li et al., 2009; Gao et al., 2018). The A–Ci curve is shown in the Supplementary Figure 2.

#### Chlorophyll Fluorescence Measurements

The fluorescence parameters were determined using the machine of CF Imager, Technologia Ltd, Colchester, United Kingdom. Following the methods of Gao et al. (2018), we selected leaves that had been adapted to the light for more than 30 min and recorded the steady-state fluorescence (F_*s*_) and then applied a flash (∼8000 μmol photons m^–2^ s^–1^) to record the maximum fluorescence under light (F_*m*_’). We maintained the leaves in the dark for 3 s, turned on the far-red light, then measured the initial fluorescence F_*o*_’ under the light. Next, the leaves were shaded and dark-adapted for more than 30 min, and the minimum (F_*o*_) and maximum (F_*m*_) chlorophyll fluorescence were recorded. Then, we calculated PSII efficiency (Φ_*PSII*_) as Φ_*PSII*_ = (F_*m*_’ – F_*s*_)/F_*m*_’, maximum fluorescence as F_*m*_ = F_*m*_ – F_0_/F_*m*_ (Genty et al., 1989), photoinhibitory quenching (qL) as qL = F_0_’/F_*s*_ × (F_*m*_’ – F_*s*_)/(F_*m*_’ – F_0_’) (Kramer et al., 2004), and the electron transport rate (ETR) as ETR = (F_*m*_’ – F_*s*_)/F_*m*_’ × PPFD × 0.85 × 0.5 (Li et al., 2009).

### Physiological Measurements and Chemical Analysis

#### N and Mg Content Determination

We used 100 mg ground dried samples to determine Mg content. The samples were extracted using 5 mL of HNO_3_ and HCl solution (1:1 v/v) for 4 h at 50°C, and then the volume was adjusted to 20 mL using ddH_2_O. Five biological repeats were conducted separately. Mg content was analyzed using the ICP-OES, Optima 8000.

Total N analyses were conducted with the method from micro-Kjeldahl (Santos and Boiteux, 2013). Dried samples (0.2 g) from flag leaves were used. Five biological repeats were performed independently.

#### Chlorophyll Content Measurements

Chlorophyll concentrations were measured spectrophoto metrically according to Arnon (1949). We used 0.05 g of fresh flag leaves and 25 mL of a mixture of acetone and absolute ethanol (volume ratio 1:1) to extract leaf pigments. The samples were placed in a 30°C incubator for about 24 h, and the extract was mixed several times. The absorbances at 470, 645, and 663 nm were measured to calculate the Chl a and Chl b concentrations, respectively. The chlorophyll concentration is the sum of Chl a and Chl b.

#### Rubisco and Rubisco Activase (RCA) Content Determination

Rubisco content was measured using SDS-PAGE following the methods of Makino et al. (1985) with modifications. We ground 10-cm^2^ frozen leaf samples and extracted each sample in 5 mL of buffer solution prepared according to the methods. Then, we centrifuge the mixture at 15,000 × *g* for 15 min at 4°C and mixed the crude enzymatic extract with 5 × loading buffer. We boiled 1 mL of the mixture for 1 min prior to electrophoresis. The amount of each sample was 10 μL used in the electrophoresis which was at 100 V for about 8 h. After that, the gels were stained with 0.25% (w/v) Coomassie Blue R-250. The gels were destained with 25% ethanol and 8% glacial acetic acid solution. Subunits of 55 and 15 kDa were put into a container with 2 mL of formamide. Then, the samples were kept in a 50°C water bath overnight. The absorbance at 595 nm (OD_595_) was measured. The calibration curve is shown in the Supplementary Figure 3.

Rubisco activase content was determined using an RCA Kit [Plant Rubisco Activase Enzyme-linked Immunoassay Assay (ELISA) Kit, Jianglai Biotechnology Co., Ltd., Shanghai] according to the manufacturer’s instructions. The double-antibody sandwich method was used to determine the RCA content of the plant according to Choi and Roh (2003) with modifications. Ten microliters of crude enzymatic extract with 40 μL sample diluent was added with 100 μl of rabbit anti-Rubisco activase antiserum in a 96-well microtiter plate, which were precoated with the RCA capture antibody. Then, the plate was incubated at 37°C for 60 min. After washing the plate five times, 100 μL peroxidase substrate was added. After mixing, the plate was kept in the dark for 15 min at room temperature. Finally, 1 M HCl was added to terminate the reaction. We measured OD_450_ within 15 min after adding the termination solution.

#### Rubisco and RCA Activity Determination

Rubisco activity was determined following the method of Sharwood et al. (2016). We measured the rate of NADH oxidation at 340 nm. The NADH-linked assays retrieve lower values of Rubisco activity compared with the ^14^CO_2_ fixation assay, but the NADH-linked assays were correlated strongly with the radiometric assay (Sales et al., 2020). NADH-linked assay is widely used in Rubisco activity, but microtiter plate-based assays may decrease the accuracy of the results (Sales et al., 2020), so we utilized cuvettes to measure the change in absorbance in a spectrophotometer. The recipes of extract buffer solution [5 ml pH 8.0 buffer solution (50 mM pH 7.5 Tris–HCl), 10 mM β-mercaptoethanol, 12.5% (v/v glycerol, 1 mM EDTA-Na_2_, 10 mM MgCl_2_, 1% (m/v) polyvinylpyrrolidone)], assay buffer solution (100 mmol EPPS-NaOH pH 8.0, 10 mmol MgCl_2_, 1 mmol EDTA, 0.2 mmol NADH, 20 mmol NaHCO_3_, 5 mmol dithiothreitol, 5 mmol ATP, 10 U⋅ml^–1^ creatine phosphokinase, 10 U⋅ml^–1^ 3-phosphoglyceric phosphokinases, 10 U⋅ml^–1^ glyceraldehyde-3-phosphate dehydrogenases, and 5 mmol phosphocreatine), and activating solution (50 mM Tris–HCl pH 7.5, 40 mM MgCl_2_, 20 mM NaHCO_3_) were according to Gao et al. (2018). We ground 10-cm^2^ frozen leaf samples and extracted each sample using 5 mL of pH 8.0 buffer solution. The mixture was centrifuged at 15,000 × *g* for 1 min at 4°C. The initial Rubisco activity was measured using a cuvette containing 100 μL of the crude enzymatic extract, 700 μL of assay buffer, and 200 μL of 10 mmol RuBP. The change in absorbance at 340 nm was monitored for 60 s (3 s per measurement) at room temperature. To determine the total Rubisco activity, 100 μL of crude enzymatic extract was activated for 10 min at 25°C in RuBP assay buffer by adding 100 μL of activating solution. Next, 100 μL of this mixture was added with 700 μL of assay buffer and 200 μL of 10 mmol RuBP sequentially, and the OD value at 340 nm was monitored for 1 min (3 s per measurement). The ratio of the initial activity over the total activity was calculated as the Rubisco activation state.

Rubisco activase activity measurements were processed following the process from Carmo-Silva and Salvucci (2011). The uncarbamylated Rubisco in the desalt extracts promoted the formation of the inactive Rubisco–RuBP complex. Frozen leaves were rapidly extracted at 4°C with 5% PEG_3350_ and 4 mM RuBP and then incubated for 5 min at 4°C. Next, the extract was added to two parallel (A and B) reactions at 25°C. The recipes of the A and B solutions were according to Carmo-Silva and Salvucci (2011). In these reactions, the RuBP concentration was 3.6 mM. Rubisco activation was tracked by evaluating Rubisco activity in aliquots taken at every half minute till 5 min after initiation of reaction. The rate of Rubisco activity was the increase in the fraction of Rubisco active sites from 1.5 to 3 min by determining the Rubisco activity in reactions without ATP.

#### ATP and ADP Content Determination

We used ATP and ADP Bioluminescence Assay Kit (Beyotime, Jiangsu, China) to determine ATP and ADP contents, respectively, following the manufacturer’s instructions. Twenty milligrams of wheat leaf samples were collected in 200 μL pre-chilled lysis buffer, and a glass homogenizer was used to fully lyse the leaf tissue. The samples were centrifuged at 10,000 × *g* for 2 min at 4°C. The supernatant was collected. The ATP detection working solution was prepared according to the kit protocol. To each well, we added 100 μL of extract, followed by 100 μL of working solution. The luciferase signals were detected for 30 s using a multifunctional microplate reader (SpectraMax M2). A standard ATP concentration curve ranging from 1 pM to 1 M was prepared by gradient dilution. ADP concentration measurements were conducted by conversion to ATP.

### Statistical Analysis

All data were analyzed using the SPSS ver. 10.0 software (SPSS Inc., United States). Two-way analysis of variance was performed for all data, and the means were compared using Duncan’s multiple-comparison tests at a significance level of *P* < 0.05. We used a minimum of three biological replicates. All graphs presented were produced using the GraphPad Prism 9 (GraphPad Software, San Diego, CA, United States), and tables were produced using Microsoft Excel 2016 (Microsoft, Redmond, WA, United States).

## Results and Discussion

### Results

#### Grain Filling Duration, Biomass, and Yield

High-temperature stress treatment significantly affected the 1000-grain weight of wheat plants, resulting in decreased yield (Table 1). The 1000-grain weight was reduced by 8.37 and 8.48% in the CK-HT treatment and by 5.01 and 5.51% in the Mg-HT treatment, during 2016–2017 and 2017–2018, respectively. Grain yield was reduced by 10.28 and 10.89% in the CK-HT treatment and by 6.6 and 7.9% in the Mg-HT treatment during 2016–2017 and 2017–2018, respectively. Mg treatment reduced the loss in grain weight and yield caused by HTS.

**TABLE 1:** Effect of Mg application on the grain filling duration (days), post-anthesis biomass (PAB, g pot^–1^), biomass at maturity (MB), 1000-kernel weight (TKW, g), and grain yield (g pot^–1^) of wheat under high-temperature stress (HTS) during grain filling.

	Treatments	Filling duration	PAB	MB	TKW	Grain yield
2016–2017	CK-OT	34.67^ab^	33.71^b^	108.5^b^	41.20^b^	43.48^b^
	CK-HT	31.33^c^	27.47^c^	102.3^c^	37.75^c^	39.01^c^
	Mg-OT	36.33^a^	36.11^a^	112.6^a^	44.43^a^	47.42^a^
	Mg-HT	34.00^b^	32.86^b^	109.3^b^	42.20^b^	44.29^b^
2017–2018	CK-OT	35.33^ab^	33.50^b^	111.1^b^	43.04^b^	47.56^b^
	CK-HT	32.00^c^	28.53^c^	106.1^c^	39.39^c^	42.38^c^
	Mg-OT	37.33^a^	37.66^a^	116.5^a^	46.28^a^	51.24^a^
	Mg-HT	34.33^b^	34.24^b^	113.0^b^	43.73^b^	47.17^b^

*CK-OT, control fertilizer with optimal temperature (OT); CK-HT, control fertilizer with high-temperature stress (HTS); Mg-OT, Mg with OT; Mg-HT, and Mg with HTS. Means were compared using Duncan’s multiple-comparison tests. Different lowercase letters after data in the same column indicate significant variations within the same year at *P* < 0.05.*

After anthesis, CK-HT and Mg-HT treatment durations were shortened by about 3 days due to HTS (Table 1). However, the duration of Mg-OT treatment was prolonged by about 3 days compared to CK-OT. As a result of HTS, biomass accumulation after anthesis declined more in CK-HT (12.91 and 16.12%, 2016–2017 and 2017–2018, respectively) than in Mg-HT treatment (8.03 and 7.92%) (Table 1). Biomass at maturity decreased under HTS treatment by 4.48 and 5.75% in CK-HT, and 2.94 and 2.89% in the Mg-HT treatment during both years (Table 1). These results indicate that Mg application enhanced biomass accumulation and reduced the loss induced by HTS.

#### Mg and N Content

**FIGURE 1:**
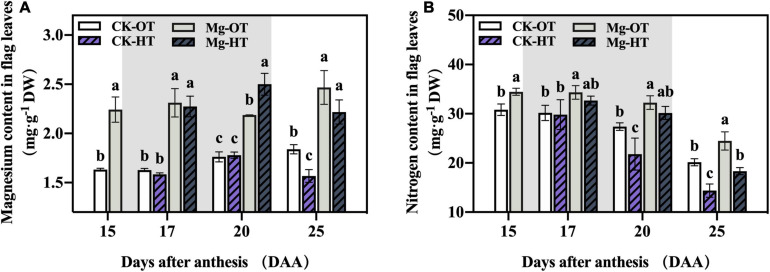
Effect of Mg application on magnesium content (mg⋅g^–1^ DW) **(A)** and nitrogen content (mg⋅g^–1^ DW) **(B)** under high-temperature stress during the grain filling. Treatments: CK-OT, control fertilizer + optimal temperature; CK-HT, control fertilizer + high temperature; Mg + OT, Mg fertilizer + optimal temperature; Mg + HT, and Mg fertilizer + high temperature. The shaded area indicates the high -temperature treatment period. Two-way analysis of variance was performed, and the means were compared using Duncan’s multiple-comparison tests. Different lowercase letters indicate significant variations within the same day at *P* < *0.05*; the error bars represent standard error (SE) of three biological replicates.

The effects of Mg application on Mg and N content in flag leaves under HTS are shown in Figure 1. Mg application promoted increases in Mg and N content in flag leaves at 15DAA. Mg and N content did not change significantly in plants under HTS (CK-HT) relative to control plants (CK-OT) at 17DAA, but a reduction in N content was observed at 20DAA. At 25DAA, Mg and N contents in flag leaves of CK-HT decreased significantly. However, in Mg-treated plants, the Mg content of flag leaves increased dramatically at 20DAA and decreased at 25DAA in Mg-HT, although these differences were not significant compared to the Mg-OT treatment. The N content of flag leaves remained stable at 20DAA and decreased significantly at 25DAA in Mg-HT plants. These results indicate that Mg treatment increased the Mg and N content of flag leaves and reduced N loss induced by HTS.

#### Photosynthesis and Related Attributes

**FIGURE 2:**
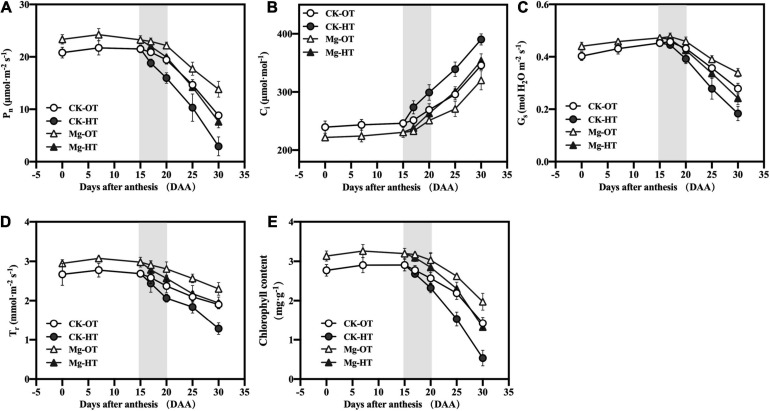
Effect of Mg application on net photosynthesis rate (P_*n*_) **(A)**, intercellular CO_2_ concentration (C_*i*_) **(B)**, stomatal conductance (G_*s*_) **(C)**, transpiration rate (T_*r*_) **(D)**, and chlorophyll content **(E)** under high-temperature stress during the grain filling. Vertical bars above mean indicate the SE of five replicates at *P* < 0.5.

The P_*n*_ of wheat flag leaves under all treatments generally decreased during growth (Figure 2). HTS decreased P_*n*_ (9.93%), transpiration rate (T_*r*_) (5.64%), leaf stomatal conductance (G_*s*_) (2.64%), and chlorophyll content (3.33%) and increased C_*i*_ (8.84%) at 17DAA, although the changes in G_*s*_, T_*r*_, and chlorophyll contents were not significant. At 20DAA, P_*n*_, G_*s*_, T_*r*_, and chlorophyll contents decreased under HTS by 17.89, 9.14, 13.11, and 9.57%, respectively, compared with CK-OT, whereas C_*i*_ increased significantly, by 11.10%. Similar trends were observed at both 25DAA and 30DAA. At 17DAA in the Mg-HT treatment, P_*n*_ (4.30%), G_*s*_ (2.08%), T_*r*_ (4.25%), and chlorophyll contents (2.63%) were decreased and Ci (2.34%) was increased, but not significantly. At 20DAA, Mg-HT treatment stabilized C_*i*_ (4.91% increase, less than CK-HT), and P_*n*_, G_*s*_, T_*r*_, and chlorophyll contents decreased by 9.92, 7.33, 8.55, and 6.10%, respectively, compared to Mg-OT. Plants were also less affected by HTS in the Mg-HT treatment than in the CK-HT treatment at 25DAA and 30DAA. The above results indicated that Mg could enhance the photosynthetic capability and stabilize that under HTS.

HTS decreased CE (27.27%) and V_*Cmax*_ (21.71%) at 17DAA in CK-HT and also decreased J_*max*_ (5.02%), but not significantly (Table 2). CE, V_*Cmax*_, and J_*max*_ decreased significantly at 20DAA and 25DAA. These findings indicate that CE and V_*Cmax*_ were inhibited earlier than J_*max*_ under HTS. At 15DAA, Mg application improved CE and V_*Cmax*_. Moreover, compared with CK-HT, V_*Cmax*_ and CE did not change significantly following Mg treatment at 17DAA under HTS. At 20DAA and 25DAA, CE, V_*Cmax*_, and J_*max*_ remained at higher levels in the Mg treatment than in CK. The difference responses between CK-HT and Mg-HT at 17DAA suggested the Mg function in the photosynthesis process under HTS.

**TABLE 2:** Effects of Mg application on carboxylation efficiency (CE), light-saturated potential rate of electron transport (J_*max*_, μmol⋅m–2⋅s–1), maximum carboxylation rate limited by Rubisco (V_*Cmax*_, μmol⋅m–2⋅s–1) under HTS during the grain filling stage.

		CE	V_*Cmax*_	J_*max*_
15DAA	CK	0.11^b^	80.93^b^	170.07^a^
	Mg	0.14^a^	91.35^a^	175.90^a^
17DAA	CK-OT	0.11^b^	76.45^b^	164.87^ab^
	CK-HT	0.08^c^	59.85^c^	156.60^b^
	Mg-OT	0.13^a^	89.82^a^	170.96^a^
	Mg-HT	0.13^a^	82.74^ab^	165.88^ab^
20DAA	CK-OT	0.10^b^	67.24^b^	153.27^a^
	CK-HT	0.07^c^	51.03^c^	129.81^c^
	Mg-OT	0.13^a^	79.96^a^	159.73^a^
	Mg-HT	0.09^b^	69.82^b^	141.66^b^
25DAA	CK-OT	0.07^b^	55.10^b^	137.26^a^
	CK-HT	0.03^c^	38.87^c^	87.03^c^
	Mg-OT	0.10^a^	68.89^a^	143.26^a^
	Mg-HT	0.06^b^	57.93^b^	105.57^b^

*Means were compared using Duncan’s multiple-comparison tests. Different lowercase letters after data in the same column indicate significant variations within the same year at P < 0.05.*

#### Light Energy Utilization and ETR

Light-dependent reactions showed that HTS did not decrease the maximum photochemical efficiency (F_*v*_/F_*m*_) at 17DAA, but Φ_*PSII*_ and ETR decreased due to a reduction in qL in the CK-HT treatment (Table 3). Higher non-photochemical chlorophyll fluorescence quenching (NPQ) was observed in CK-HT at 17DAA. Φ_*PSII*_, qL, F_*v*_/F_*m*_, and ETR decreased significantly at 20DAA and 25DAA, and NPQ increased. Mg-treated plants showed significantly higher Φ_*PSII*_, qL, and ETR at 15DAA, which was consistent with P_*n*_ and its attributes. Φ_*PSII*_, qL, ETR, and F_*v*_/F_*m*_ were reduced at 20DAA and 25DAA in both CK-HT and Mg-HT, but smaller reductions were observed in Mg-HT plants. A small reduction in NPQ was also observed in Mg-treated plants under HTS. These results indicated that compared with the control, Mg-treated plants had a stronger light energy utilization ability and maintains electron transfer under HTS.

**TABLE 3:** Effects of Mg application on actual photochemical efficiency of PSII in the light (Φ_*PSII*_), photochemical quenching (qL), the maximum quantum efficiency of PSII (F_*v*_/F_*m*_), electron transport rate (ETR), and non-photochemical quenching (NPQ) under HTS during the grain filling stage.

	Treatment	Φ _*PSII*_	qL	ETR	NPQ	F_*v*_/F_*m*_
DAA15	CK	0.39^b^	0.33^b^	131.71^b^	1.89^a^	0.82^ab^
	Mg	0.42^a^	0.38^a^	140.22^a^	1.91^a^	0.84^a^
DAA17	CK-OT	0.38^b^	0.29^b^	126.22^b^	1.96^b^	0.83^a^
	CK-HT	0.32^c^	0.24^c^	107.52^c^	2.37^a^	0.82^ab^
	Mg-OT	0.40^a^	0.32^a^	134.18^a^	1.94^b^	0.83^a^
	Mg-HT	0.39^a^	0.31^a^	131.41^a^	2.05^b^	0.83^a^
DAA20	CK-OT	0.35^b^	0.28^b^	118.33^b^	2.01^c^	0.81^a^
	CK-HT	0.30^c^	0.22^c^	100.62^c^	2.74^a^	0.76^c^
	Mg-OT	0.38^a^	0.32^a^	128.10^a^	2.03^c^	0.83^a^
	Mg-HT	0.34^b^	0.28^b^	115.08^b^	2.36^b^	0.79^b^
DAA25	CK-OT	0.24^b^	0.26^b^	79.18^b^	2.35^bc^	0.76^b^
	CK-HT	0.16^c^	0.15^d^	54.99^c^	3.48^a^	0.73^c^
	Mg-OT	0.30^a^	0.30^a^	102.14^a^	2.03^c^	0.78^a^
	Mg-HT	0.26^b^	0.21^c^	87.14^b^	2.73^b^	0.75^b^

*Means were compared using Duncan’s multiple-comparison tests. Different lowercase letters after data in the same column indicate significant variations within the same day (*P* < 0.05, *n* = 5).*

#### Rubisco Content and Activation State

**FIGURE 3:**
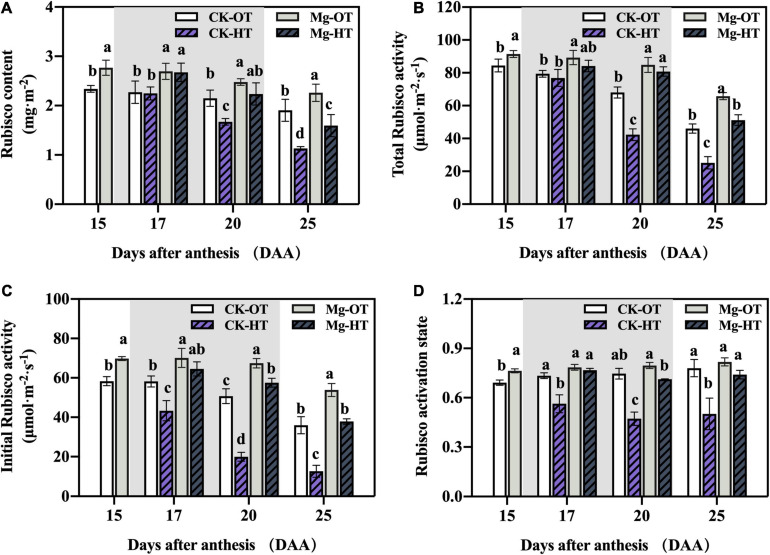
Effect of Mg application on Rubisco content and activity under HTS during the grain filling stage. Two-way analysis of variance was performed; each bar is the mean ± SE of five replications. Different lowercase letters indicate significant variations within the same day at *P* < 0.5 (Duncan’s multiple-comparison tests). **(A)** Rubisco content, **(B)** Total Rubisco activity, **(C)** Initial Rubisco activity, **(D)** Rubisco activation state.

Rubisco content and total activity decreased significantly under HTS at 20DAA and 25DAA (Figures 3A,B), but they did not change significantly at 17DAA. HTS led to significantly decreased initial activity at 17DAA, 20DAA, and 25DAA (Figures 3C,D). Mg-OT plants showed higher Rubisco content (Figure 3A), total and initial activity (Figures 3B,C), and activation state (Figure 3D) compared to CK-OT at 15DAA. Rubisco content, total activity, and initial activity were higher in Mg-OT plants than CK-OT at 17DAA, 20DAA, and 25DAA. Interestingly, the initial Rubisco activity and the activation state were maintained in Mg-treated plants relative to CK-HT plants at 17DAA, although decreasing trends were observed in Mg-HT plants at 20DAA and 25DAA. Mg-HT plants showed a smaller decrease in initial Rubisco activity and activation state than CK-HT plants at 20DAA and 25DAA. This result was consistent with the result in Table 2.

#### RCA Concentration and Activity

**FIGURE 4:**
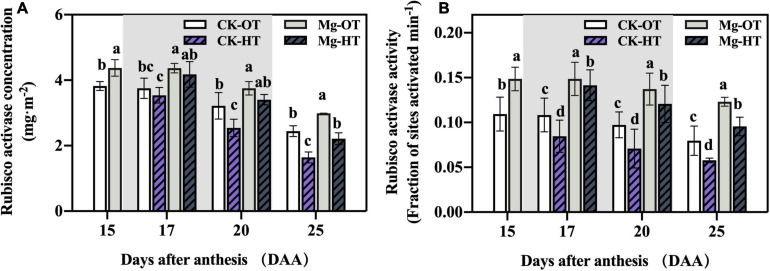
Effect of Mg application on Rubisco activase concentration and activity under HTS during the grain filling stage. Two-way analysis of variance was performed; each bar is the mean ± SE of five replications. Different lowercase letters indicate significant variations within the same day at *P* < 0.5 (Duncan’s multiple-comparison tests). **(A)** Rubisco activase concentration, **(B)** Rubisco activase activity.

The initial Rubisco activity and activation state depend on RCA activation. In our study, the total RCA pool was measured without distinguishing isoforms. The RCA concentration did not change significantly at 17DAA but decreased significantly in the CK-HT treatment at 20DAA and 25DAA (Figure 4A) and in the Mg-HT treatment at 25DAA. This result was consistent with our Rubisco content results. RCA activity in the Mg treatments was significantly higher than in CK at all stages (Figure 4B). Interestingly, HTS decreased RCA activity significantly at 17DAA in CK-HT but did not significantly affect that in the Mg-HT treatment. However, RCA activity decreased significantly under HTS in both CK and Mg treatments at 20DAA and 25DAA.

#### ATP and ADP Content and ATP/ADP

**FIGURE 5:**
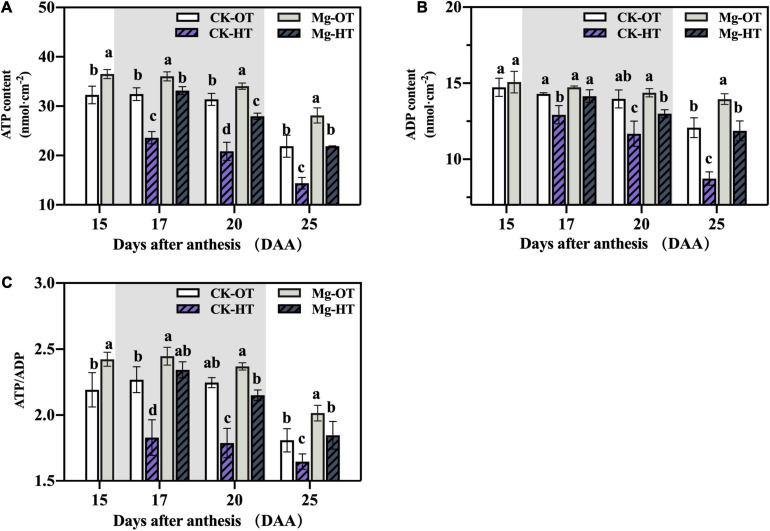
Effects of Mg application on ATP content, ADP content, and ATP/ADP in flag leaves of wheat under HTS during grain filling. Two-way analysis of variance was performed; each bar is the mean ± SE of five replications. Different lowercase letters indicate significant variations within the same day at *P* < 0.5 (Duncan’s multiple-comparison tests). **(A)** ATP content, **(B)** ADP content, **(C)** ATP/ADP.

Mg application increased the ATP content and ATP/ADP ratio compared with CK in the OT treatment (Figure 5). However, there was no significant variation in ADP content between CK and Mg. HTS inhibited ATP synthesis, whereas Mg-HT sustained the ATP synthesis and high ATP/ADP ratios. The reduction in the content in ATP corresponded to the electron transfer rate. ATP and ADP content and the ATP/ADP ratio decreased under HTS at 20DAA and did not recover at 25DAA, indicating that the plants had reached senescence at 25DAA. Together, these results indicate that HTS inhibits ATP and ADP production and decreases the ratio of ATP over ADP but that Mg alleviates these losses.

#### Correlations Among the Rubisco Activities, RCA Activity, and ATP/ADP Ratio

**FIGURE 6:**
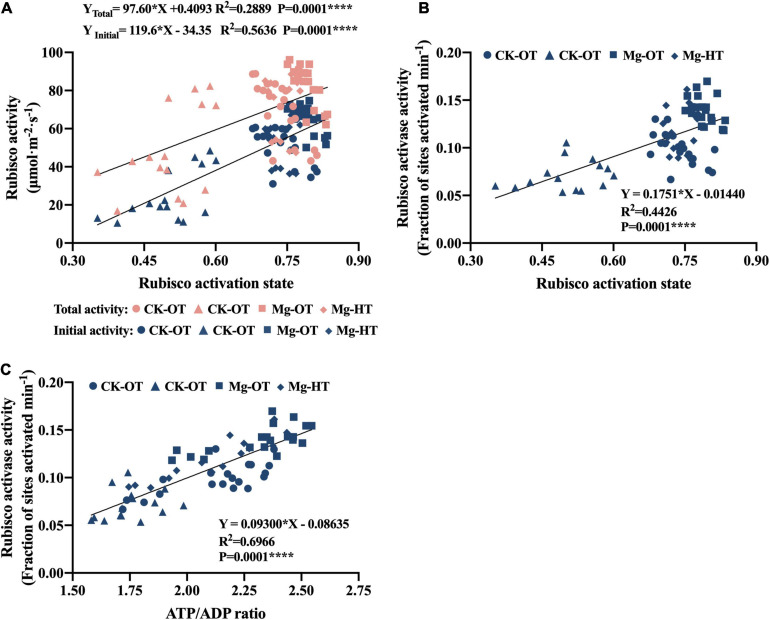
Relationships between Rubisco activities, RCA activity, ATP/ADP ratio, and Rubisco activation state. Values of Rubisco activities, RCA activity, ATP/ADP ratio, and Rubisco activation state are from individual sample values in Figures 3–5 (*n* = 70). “●” Represents values of CK-OT, “▲” represents CK-HT, “■” represents Mg-OT, and “◆” represents Mg-HT. The asterisks represent the statistical significance at the *P* level shown in the figure, respectively. **(A)** ATP content. **(B)** ADP content. **(C)** ATP/ADP.

The initial Rubisco activity and the total Rubisco activity were positively correlated with Rubisco activation state (Figure 6A), while the initial activity contributed more to increased Rubisco activation state than to total Rubisco activity. The RCA activity was also positively correlated with the activation state and the ATP/ADP ratio (Figures 6B,C). This implied that a higher ATP/ADP ratio was closely related to the enhancement of the RCA activity, and then the activation state was also improved by higher Rubisco initial activity and RCA activity.

### Discussion

#### Yield

Global warming is a significant cause of HTS, which poses a serious threat to wheat production in many countries, especially during the reproductive and grain filling phases (Farooq et al., 2011; Khan et al., 2020). Wheat yield reduction under HTS is correlated with fewer spikes and smaller grains (Gibson and Paulsen, 1999; Lesk et al., 2016), whereas grain weight is significantly reduced by high temperatures in the middle and late stages of grain filling (Tahir and Nakata, 2005; Wajid et al., 2018). Assimilate transport from flag leaf to grain is substantially reduced by temperatures above 30°C (Plaut et al., 2004). In this study, HTS shortened the duration of the post-anthesis stage and decreased biomass accumulation (Table 1), seed weight, and grain yield. It has been proposed that grain weight was sensitive to the amount of the Mg in the soil (Potarzycki, 2008; Szulc, 2010; Grzebisz, 2013). In this study, the application of Mg fertilizer promoted crop growth and yield formation, which has also been alleviated under HTS. Leaf symptoms in Mg-deficient plants are similar to those of senescent leaves but can be recovered by sufficient Mg application (Uchida, 2000; Tanoi and Kobayashi, 2015). In this study, Mg application extended the duration of the grain filling stage and produced more biomass after anthesis under HTS, leading to higher grain weight and yield formation.

#### Mg and N Content

Under normal conditions, Mg content accounts for 0.15–0.35% of the dry weight of vegetative plant parts (Marschner, 1995; Tanoi and Kobayashi, 2015). During early vegetative growth, the critical whole-shoot Mg concentration is 0.1% for wheat (Jones and Wolf, 1991). In this study, Mg-treated plants alleviated yield loss under HTS and increased yield under normal temperature conditions, perhaps because Mg concentrations in the flag leaves of Mg-treated plants were at adequate levels (Mengutay et al., 2013), whereas CK plants were near the threshold of Mg deficiency. The most significant change during senescence is the breakdown of chloroplasts, which account for more than 70% of total N (Bascunan-Godoy et al., 2018). The rate of senescence and the remobilization of leaf N are related to the plant N nutrition status (Hortensteiner and Feller, 2002; Nehe et al., 2020). Previous studies have suggested that Mg application supports N uptake (Peng et al., 2019). Our findings also demonstrated that Mg promotes N accumulation, which were consistent with those of Peng et al. (2019). Additionally, HTS accelerates N degradation in flag leaves, whereas Mg stabilized N content, which suggests that Mg alleviates senescence in flag leaves under HTS during the filling stage.

#### Photosynthesis and Related Attributes

Photosynthesis is pivotal to crop yield production, and photosynthetic capacity decreases gradually during plant senescence. Leaf photosynthesis is the physiological process that is most sensitive to HTS (Sabater and Martín, 2013), which significantly affects P_*n*_, G_*s*_, T_*r*_, and C_*i*_ (Greer and Weedon, 2012; Jahan et al., 2019). Reduced P_*n*_ under HTS appears to be caused by non-stomatal factors, because the stomata remain open and C_*i*_ is not reduced when the photosynthetic structure is damaged (Mathur et al., 2014). In this study, G_*s*_ and T_*r*_ were not inhibited, but P_*n*_ decreased at 17DAA, indicating that the reduction in P_*n*_ under HTS in the early stages was not due to stomatal factors. Stabilization of leaf transpiration may be a way for wheat to improve leaf overheating (Yang et al., 2012). However, C_*i*_ increased significantly, indicating that HTS reduced CO_2_ utilization efficiency. Mg treatment improved P_*n*_ prior to HTS treatment. P_*n*_ has been found to be higher in Mg-sufficient plants than in Mg-deficient plants (Laing et al., 2000). In this study, P_*n*_ and T_*r*_ were stabilized in Mg-treated plants at 17DAA under HTS, compared with the CK. Similar Mg effects have been reported in *Torreya grandis* seedlings under toxic lead (Pb^2+^) stress (Shen et al., 2016). Mg application also kept high levels of G_*s*_, which may be attributed to Mg playing a role in the regulation of anion–cation balance and cell turgor in cells along with K (Gerendás and Führs, 2013; Hasanah, 2018). In this study, photosynthetic capacity gradually decreased with wheat leaf senescence, and its inhibition by HTS was more significant at 25DAA and 30DAA, even in the Mg treatments, indicating that HTS irreversibly accelerates senescence during the grain filling stage in wheat.

The factors directly limiting photosynthesis at different degrees of HTS remain controversial because the photosynthetic system produces varying responses at different temperature ranges (Salvucci and Crafts-Brandner, 2004b; Yamori et al., 2013). CE, V_*Cmax*_, and J_*max*_ decreased under HTS (Weston and Bauerle, 2007; Ferguson et al., 2020). V_*Cmax*_ decreased earlier than J_*max*_ at 17DAA, whereas both decreased synchronously at 20DAA and 25DAA; thus, J_*max*_ decreased after V_*Cmax*_ inhibition. Under Mg application, V_*Cmax*_ did not decrease at 17DAA, indicating that Mg stabilized Rubisco carboxylation capacity under HTS. Even at 20DAA and 25DAA, these levels were higher under Mg treatment; thus, HTS may directly inhibit Rubisco carboxylation activity and electron transfer during the photosynthetic light reaction, whereas Mg stabilizes V_*Cmax*_ under short-term HTS, promoting carbon reactions.

#### Light-Dependent Reaction

The light system of plant leaves absorbs light energy and converts it into ATP and NADPH (Ashraf and Harris, 2013). When these processes are impaired, electron leakage damages the chloroplast structure (Wang et al., 2015). F_*v*_/F_*m*_ is a measure of photosynthetic capacity and can be used to verify the integrity of the PSII (Gregersen et al., 2014). In this study, under short-term HTS (17DAA), the F_*v*_/F_*m*_ of the light reaction system did not change, indicating that the photosynthetic light system of wheat leaves was not damaged under HTS, and the light reaction center retained its ability to self-regulate the photosynthetic system. This finding is consistent with our J_*max*_ results. HTS also led to PSII closure in control plants by decreased qL and increased NPQ, which is a strategy for protecting the photosynthetic system under stress conditions (Maxwell and Johnson, 2000). However, PSII remained open in Mg-treated plants under HTS at 17DAA, and higher ETR and F_*v*_/F_*m*_ values were observed at 20DAA and 25DAA, indicating that Mg application under HTS in the grain filling stage promotes light energy utilization and electron transfer, which are beneficial to the synthesis of ATP and NADPH.

#### Rubisco Activation

Photosynthesis inhibition in wheat leaves under HTS is mainly caused by decreased Rubisco carboxylation activity (Scaligero, 2004; Sharwood et al., 2016; Scafaro et al., 2019; Degen et al., 2020, 2021). However, Rubisco is actively degraded during leaf senescence (Imai et al., 2008). In this study, higher Rubisco content was found in Mg-treated plants, which may be related to the uptake of N by applying Mg (Peng et al., 2019), and the Rubisco content is positively correlated with leaf-N content (Makino, 2003). Also, previous studies indicated that total Rubisco activity increased linearly with increasing leaf N (Cheng and Fuchigami, 2000). Therefore, the loss of the total Rubisco activity under HTS at 20DAA and 25DAA may be due to the reduction in the Rubisco amounts caused by accelerated senescence in the flag leaves. Mg stabilized the N content in the leaves, which alleviated the loss of Rubisco amount as well as the total Rubisco activity. However, in this study, under short-term HTS (17DAA), Rubisco content and total activity did not significantly decrease, whereas initial Rubisco activity and activation state were inhibited; this result suggests that Rubisco activation state and activity are sensitive to HTS. Various studies indicated that the decrease in the Rubisco activation state was the main cause of photosynthetic inhibition under moderate heat stress (Salvucci and Crafts-Brandner, 2004a; Degen et al., 2021) and a decrease in the activation state of Rubisco was thought to reflect a loss of carbamylation due to changes in stromal pH and Mg^2+^ concentration (Weis, 1981). In the present study, the initial Rubisco activity decreased earlier at 17DAA and decreased more at 20DAA compared with the total Rubisco activity, which is the reason for the reduction in the Rubisco activation state under HTS. Moreover, previous study has proposed that the decrease in Mg^2+^ level in chloroplast caused a significant reduction in Rubisco activity (Lasa et al., 2000). The Rubisco activation obviously depends on the Mg^2+^ concentration (Tcherkez, 2013). In the chloroplast, where Mg^2+^ total concentration is around 10–20 mM, the RuBP concentration at least twice as high as the concentration in Rubisco sites leads to an activation level of about 90% (Von Caemmere, 1985; Tcherkez, 2013). In our results, Mg treatment significantly increased Rubisco activation state by increasing Rubisco activity, and the Rubisco activation state was stabilized under HTS at 20DAA and 25DAA. Further, in the analysis shown in Figure 6, a significant loss of the Rubisco amount caused reductions in the total Rubisco activity at 25DAA, and the initial Rubisco activity decreased significantly at that time. In contrast to previous observations (Perdomo et al., 2017; Degen et al., 2021), Rubisco activation state and Rubisco content were positively correlated, possibly due to the interplay between heat stress and the onset of leaf senescence. The Rubisco activation state and the total Rubisco activity were decreased synchronously after HTS during the late senescence stage. However, higher initial activity was more closely related to improvements in activation state.

Previous studies have shown that Rubisco enzymes bind easily with sugar phosphate or other substances, which inhibit its function (Parry et al., 2008, 2012; Scafaro et al., 2019; Degen et al., 2020, 2021). RCA and ATP can relieve Rubisco enzyme activity inhibition; therefore, RCA activity is important for stabilizing Rubisco activation (Parry et al., 2008). RCA is directly influenced by temperature (Carmo-Silva and Salvucci, 2012), and it showed the same trend between photosynthetic rate and RCA activity under HTS (Law and Craftsbrandner, 1999). The RCA activity is closely related to the heat tolerance level of wheat (Kumar et al., 2019). In the present study, HTS decreased RCA activity and concentration, but Mg treatment significantly increased RCA activity, which is consistent with the findings of previous studies (Liang et al., 2008; Kuriata et al., 2014; Hazra et al., 2015). Additionally, the RCA activity was also positively correlated with the Rubisco activation state (Figure 6B). Together, our results suggest that Mg promotes RCA activity, thereby stabilizing Rubisco activation under HTS during the wheat grain filling stage.

#### Carboxylation and Light-Dependent Reactions Are Interrelated

The CO_2_ assimilation reactions (Calvin–Benson cycle) consume ATP and NADPH to regenerate NADP^+^ and ADP, reducing the light-dependent reaction and accepting transferred electrons during stable forward photosynthesis (Parry et al., 2008). Under HTS, ATP and ADP contents decreased, indicating the blockage of photoreaction ATP synthesis. Mg-treated plants had higher ATP content, which is consistent with the findings from Busch and Ninnemann (1997). We monitored RCA activity in terms of the ratio of ATP to ADP (Carmo-Silva et al., 2015). The positive correlation between the RCA activity and the ATP/ADP ratio implied that a higher ATP/ADP ratio was closely related to the enhancement of the RCA activity (Figure 6C). HTS directly inhibited Rubisco activity, which influenced NADP^+^ and ADP reduction. Next, electron transfer was inhibited, which led to photoinhibition (Parry et al., 2008; Carmo-Silva and Salvucci, 2011; Mathur et al., 2014). Therefore, the photosynthetic system was damaged by an increase in redundant electrons. At 25DAA, ATP, and ADP contents remained higher in Mg-treated plants than in CK plants, and ATP/ADP increased significantly, indicating that Rubisco activation was maintained through Mg application, such that light energy utilization and electron transfer were relatively stabilized during the light reaction.

## Conclusion

The results of the present study indicate that HTS caused a decrease in Rubisco carboxylation activity which inhibited photosynthesis during the wheat senescence stage, whereas Mg application maintained Rubisco carboxylation by enhancing its activation state and stabilizing the electron transfer rate. Thus, photosynthesis was sustained by Mg application under HTS. These results suggest that Mg plays an indispensable role in sustaining photosynthesis during grain filling by improving Rubisco activation state under HTS conditions.

## Data Availability Statement

The original contributions presented in the study are included in the article/Supplementary Material, further inquiries can be directed to the corresponding author/s.

## Author Contributions

YS, JH, CS, ZT, and TD conceived and designed the experiments. YS, JH, CS, SXL, and SYL developed methodologies. LG, SYL, JH, and JG analyzed the experimental data. SYL, LG, AU, YS, and XL wrote the manuscript. SXL, LG, AU, DJ, WC, ZT, and TD revised the manuscript. All the authors contributed to the article and approved the submitted version.

## Conflict of Interest

The authors declare that the research was conducted in the absence of any commercial or financial relationships that could be construed as a potential conflict of interest.
