# Supplementary material for: Magnesium Application Promotes Rubisco Activation and Contributes to High-Temperature Stress Alleviation in Wheat During the Grain Filling
Source: Front Plant Sci. 2021 Jun 11;12:675582. doi: 10.3389/fpls.2021.675582 (PMC8231710; doi:10.3389/fpls.2021.675582)
Supplement: Supplementary file 1 [file Data_Sheet_1.docx]

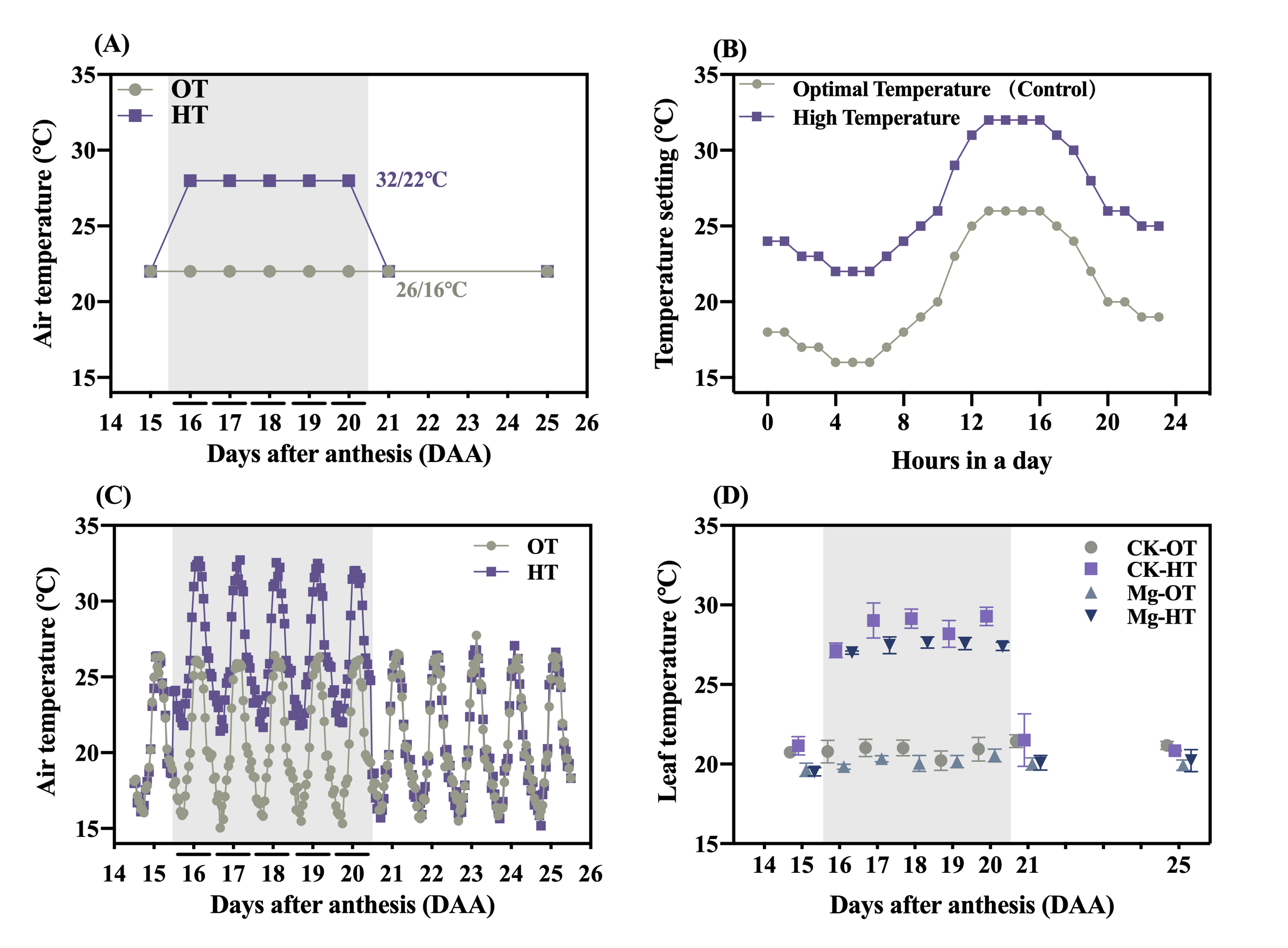
FugureS1.Air and leaf temperature during treatments days. A: Air average temperature in treatment days. B: Temperature setting in a day. C: Air actual temperature during treatment days and after treatment days. D: Leaf temperature during treatment days.


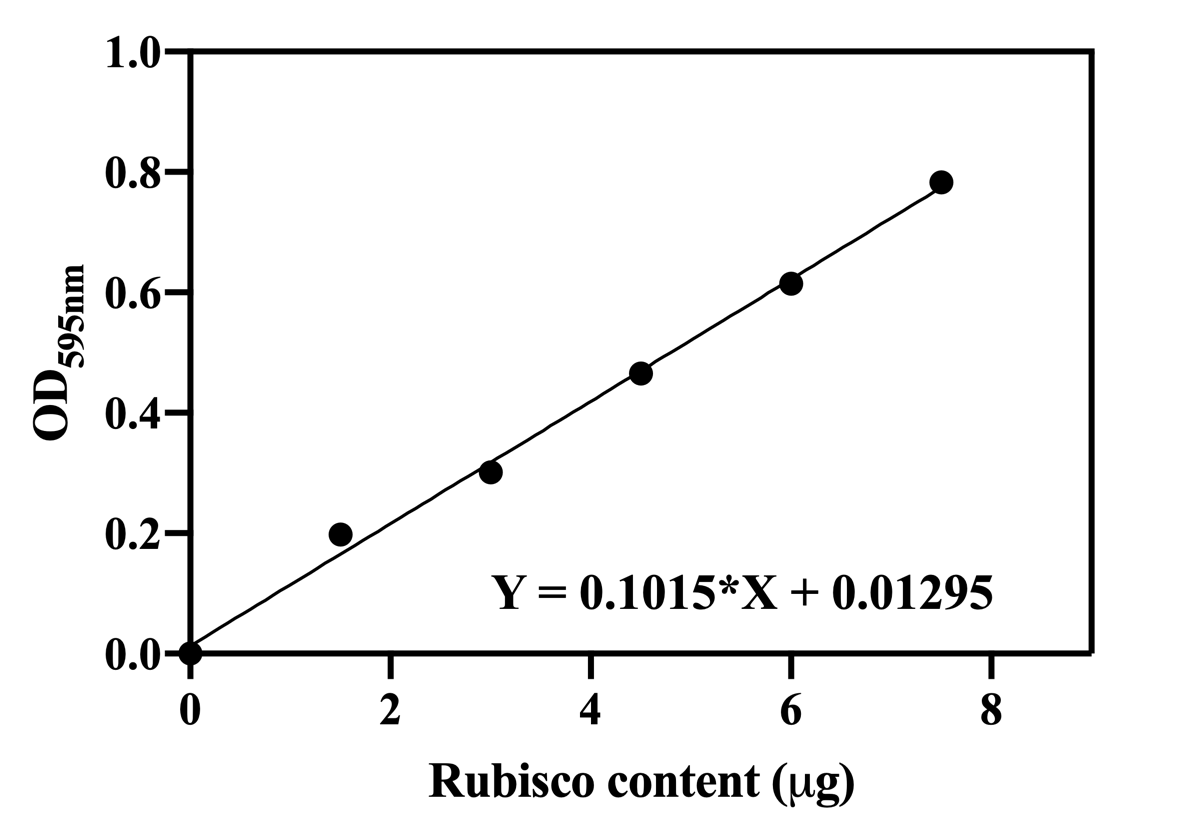

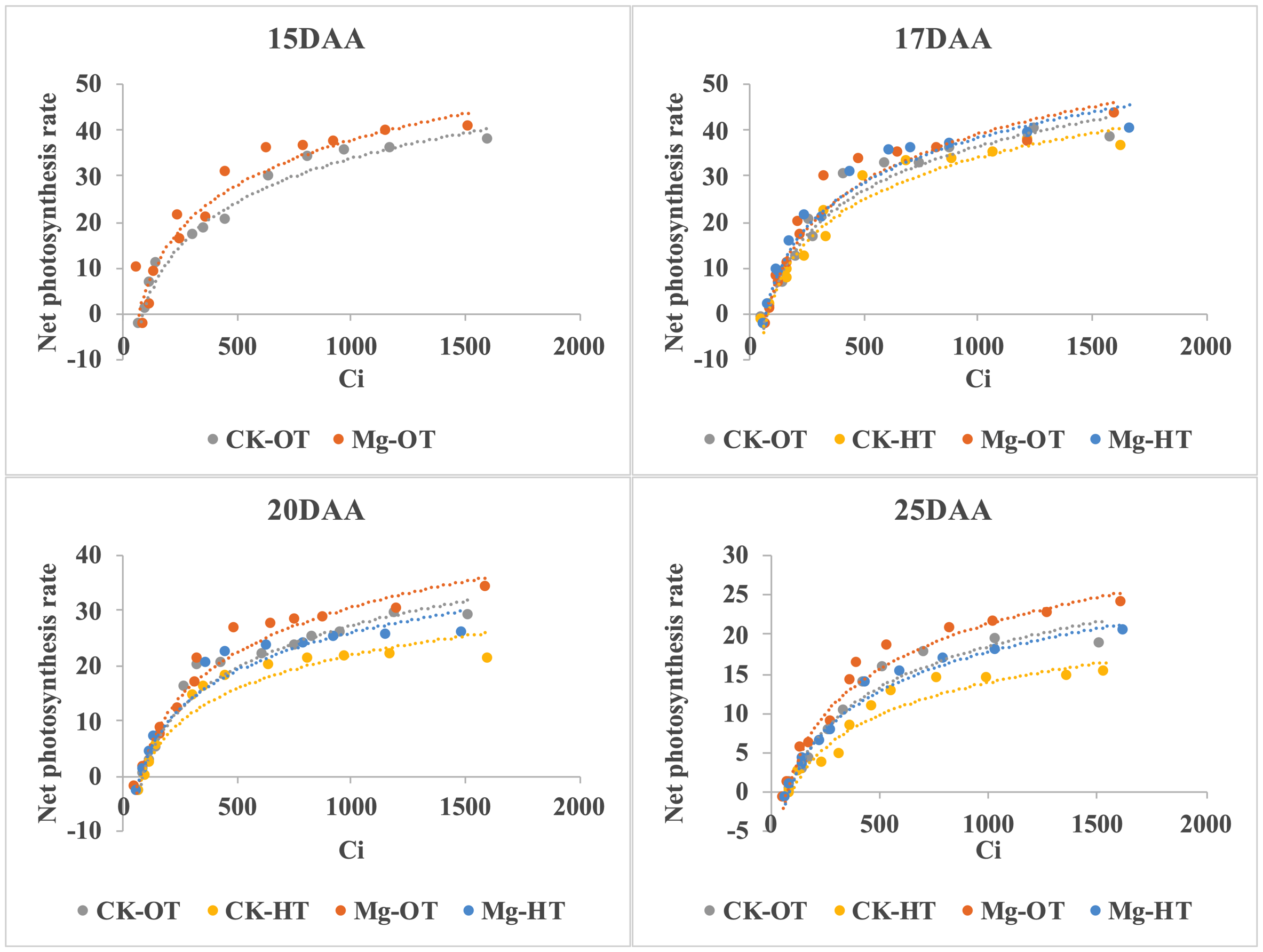
Figure S2. A-Ci curve.

Figure S3. Rubisco content calibration curve.
